# Host Tropism and Structural Biology of ABC Toxin Complexes

**DOI:** 10.3390/toxins16090406

**Published:** 2024-09-19

**Authors:** Cole L. Martin, John H. Hill, Stephen G. Aller

**Affiliations:** 1 Graduate Biomedical Sciences Pathobiology, Physiology and Pharmacology Theme, University of Alabama at Birmingham, Birmingham, AL 35294, USA; martin94@uab.edu; 2 Department of Pharmacology and Toxicology, University of Alabama at Birmingham, Birmingham, AL 35294, USA; jhh1492@uab.edu

**Keywords:** ABC toxins, translocase, cryo-EM, host tropism, structural biology, X-ray crystallography, insecticide, biotechnology

## Abstract

ABC toxin complexes are a class of protein toxin translocases comprised of a multimeric assembly of protein subunits. Each subunit displays a unique composition, contributing to the formation of a syringe-like nano-machine with natural cargo carrying, targeting, and translocation capabilities. Many of these toxins are insecticidal, drawing increasing interest in agriculture for use as biological pesticides. The A subunit (TcA) is the largest subunit of the complex and contains domains associated with membrane permeation and targeting. The B and C subunits, TcB and TcC, respectively, package into a cocoon-like structure that contains a toxic peptide and are coupled to TcA to form a continuous channel upon final assembly. In this review, we outline the current understanding and gaps in the knowledge pertaining to ABC toxins, highlighting seven published structures of TcAs and how these structures have led to a better understanding of the mechanism of host tropism and toxin translocation. We also highlight similarities and differences between homologues that contribute to variations in host specificity and conformational change. Lastly, we review the biotechnological potential of ABC toxins as both pesticides and cargo-carrying shuttles that enable the transport of peptides into cells.

## 1. Introduction

**Figure 1:**
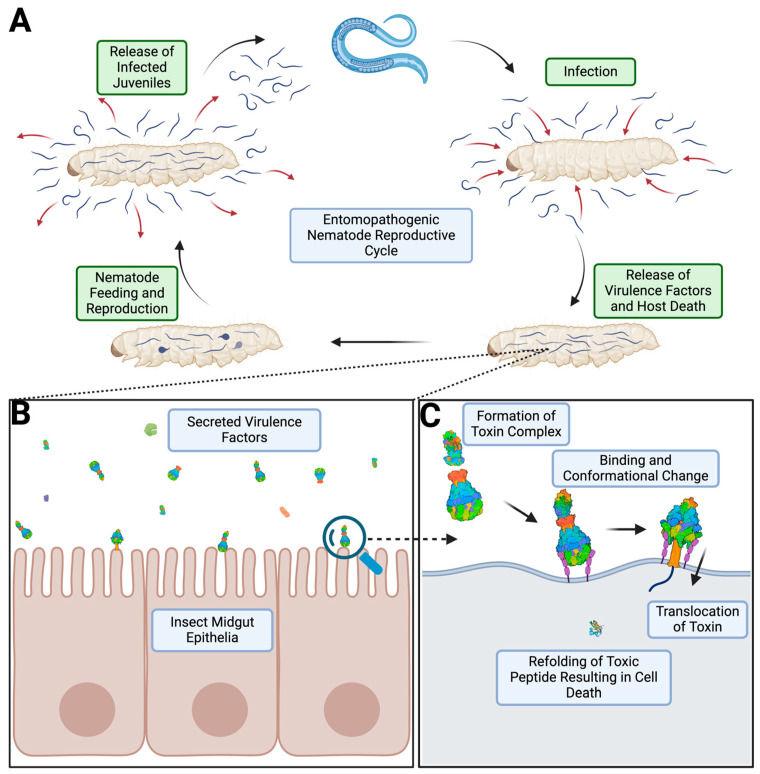
Life cycle of entomopathogenic nematodes and simplified view of ABC Tc intoxication. (**A**) Entomopathogenic nematode reproductive cycle starting from a juvenile nematode all the way through reproduction and release of offspring. (**B**) Zoomed in view of the larvae midgut epithelial cell being exposed to various virulence factors released by the bacterial symbiont of nematodes. (**C**) Simplified mechanism of ABC Tc intoxication of cells. Note: Direct injection mechanism is depicted for simplicity, although an alternative endosomal mechanism has been proposed. This figure was produced in Biorender.

ABC toxin complexes (Tcs) are a class of protein toxin translocases naturally secreted in Gram-negative and Gram-positive bacteria [1]. They were first classified in *Photorhabdus luminescens* of the family Morganellaceae [2,3]. Since this discovery, Tc subunits have been identified across 1421 bacterial genomes, including *Xenorhabdus nematophila*, *Morganella morganii*, and *Yersinia entomophaga* [4,5,6,7]. Over time, these bacteria have developed mutualistic relationships with various entomopathogenic nematodes to ensure the survival of both organisms [5,8]. In nature, these bacteria reside within the nematode digestive tract, where they are released after the nematode burrows into the host insect larvae [5] (Figure 1A). Once inside the hemocoel, the bacteria release a host of virulence factors, including ABC toxins that have been shown target insect brush border membranes of the midgut epithelia, resulting in the death of the organism for nutrient consumption and nematode reproduction [5,9,10,11,12] (Figure 1B,C).

ABC Tcs generally contain three subunits that are pathogenic to various organisms such as insects and humans [6,9,13,14,15]. The A subunit or TcA is the largest subunit, generally comprising ~80% of the full complex [16,17,18]. The TcA contains multiple domains that include IgG-like receptor binding domains (RBDs), an α-helical domain, an α-pore-forming domain, a neuraminidase-like domain, and a TcB-binding domain [19,20,21]. Of all the subunits, TcAs have been characterized most thoroughly and mediate host tropism as well as translocation of the toxic peptide across lipid bilayers [19,21,22,23]. The B subunit (TcB) is the second largest subunit of the complex, comprising ~11% of the full tripartite complex [16,24]. This subunit links the TcA to the C subunit (TcC) and controls the gating mechanism that allows the toxic cargo in TcC to be linearized into the pore-forming domain of the TcA [1,25,26]. The TcC is the smallest subunit, comprising ~9% of the full complex [1,16,26]. This subunit houses the toxic peptide, such as an ADP-ribosyltransferase or RhoGTPase, which undergoes enzymatic cleavage, initiating translocation through the TcB and TcA subunits [25,26]. These toxic peptides reside within the C-Terminal Region (C^CTR^) and undergo auto-proteolytic cleavage before translocation [27,28,29]. Once translocated inside the cell, the toxic peptide is refolded, resulting in actin polymerization, detachment of the cell cytoskeleton, and cell death [27,28,29]. Together, these subunits form a complete heterotrimeric complex, termed the ABC Tc.

**Figure 2:**
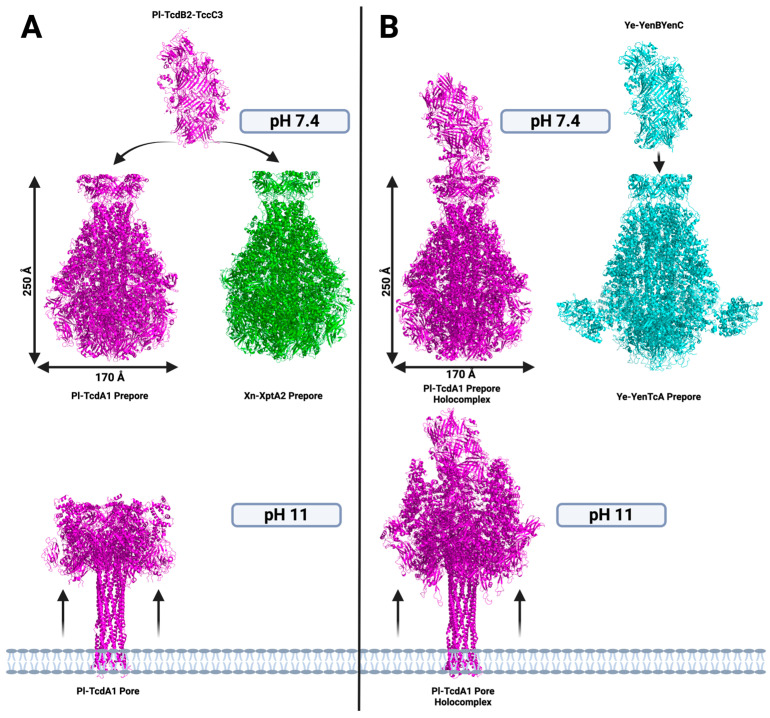
Schematic of toxin complex formation and pre-pore to pore state transition of TcAs. (**A**) Structures of the Pl-TcdA1 and Xn-XptA2 in the pre-pore state showing that the full complex with Pl-TcdB2-TccC3 can be formed between both TcAs and Pl-TcdA1 in the pore state after exposure to basic conditions. (**B**) Structures of Pl-TcdA1-TcdB2-TccC3 complex in the pre-pore state (neutral pH) and pore state (basic pH) and Ye-YenTcA, which has been shown to form a complex with Ye-YenB-YenC.

Most ABC Tcs share high sequence and structural similarity, suggesting that the basic toxin translocation mechanisms within Tcs are analogous. In the most characterized TcA, *Photorhabdus luminescens*-TcdA1 W14 (hereby referred to as Pl-TcdA1), a significant conformational change is observed when Pl-TcdA1 is exposed to extreme changes in pH, such as acidic (pH 4) and alkaline (pH 11) conditions [19,20] (Figure 2). At neutral pH, Pl-TcdA1 remains in the pre-pore state, retaining the balloon-like structure, although slight variations in pH induce subsets of the pre-pore Pl-TcdA1 to undergo conformational change into the pore state [20]. The strongly two-state conformational nature of *Pl-*TcdA1 has prompted this specific ortholog to be dubbed a binary toxin [17]. The pre-pore to pore state conformational change is characterized by the opening of the outer loops of the neuraminidase-like domains shielding the top of the hydrophobic residues of the translocation channel [18]. Subsequently, the linker region, connecting the α-helical domain to the α-pore-forming domain, entropically collapses, pulling the outer shell of the TcA into the pore state, exposing the translocation channel to the membrane [19,21]. Once the hydrophobic residues within the loop region of the translocation channel are in close proximity to a membrane, it is energetically favorable for these residues to pierce through the lipid bilayer [20] (Figure 2). After this occurs, a pore is formed across the bilayer and the toxic payload is translocated into the cell [23]. It is still poorly understood whether this translocation event happens directly across the plasma membrane of the cell or if it takes place after endocytosis and exposure to the acidic conditions of the lysosome, although this could vary between Tc homologues [12,19,22,29]. Alternatively, pH-resistant TcAs have been observed, indicating that a pH-dependent mechanism is not conserved among all Tcs [21].

The study of ABC Tcs has accelerated recently due to the potential impact this class of toxins could have on agriculture [6,9,30,31,32]. Additionally, the unique nature of these toxins’ ability to translocate peptides across a lipid bilayer has garnered attention for potential biotechnology and clinical applications [28,33,34,35]. Clinical interest also stems from the necessity of understanding the mechanism of Tcs that are pathogenic to mammals [7,36,37]. Although much has been discovered regarding the structures and mechanism of Tc translocation, there are still many unanswered questions in the field that need to be addressed. To our knowledge, the specific RBDs responsible for host tropism remain unclear. Moreover, the driver of conformational change is not uniform across all Tcs, and only one high-resolution pore state structure has been resolved [19]. Furthermore, only one high-resolution heterotrimeric holotoxin structure of a native Tc has been solved at high resolution [26]. This review serves to highlight the current literature, consolidate what is known, and to identify the gaps in knowledge concerning ABC Tc biology.

## 2. Sequence Analysis and Phylogeny of Tcs

Most bacterial genomes are often composed of a “stable” region (encoding essential cellular functions) and a “fluid” region containing plasmids, insertion sequences, prophages, and large unstable regions deemed genomic islands (GEIs). When these GEIs encode the virulence factors of pathogenic bacteria, they are referred to as pathogenicity islands (PAIs) [38]. PAIs received particular interest after it was shown that pathogenic and non-pathogenic strains of *E. coli* share less than 40% sequence identity, highlighting the role and magnitude of horizontal gene transfer in microbial evolution [39]. An extensive profiling into the *Photorhadbus luminesces* strain W14 revealed such an area, termed toxin complex island d (tcd), which was extensively mapped out and shown to be capable of encoding multiple versions of homologous TcA and TcB components [40]. To date, four total loci, or pathogenicity islands, have been established as Tca, Tcb, Tcc, and Tcd in *Photorhabdus* [18,41]. Notably, the *tcc* and *tca* loci encode multiple open reading frames (ORFs), leading to the production of several components per loci, while *tcb* and *tcd* constitute a singular ORF [3,13,42]. Regarding *tca*, *tcb*, and *tcc* loci, it has been reported that three basic types of genetic elements have been identified: the tcdA-like element, the tcdB-like element, and the tccC-like element [43]. Furthermore, given the close link between *tcc* elements/*tc* loci and their relationship to rearrangement hot spots (rhs), *tcc* elements could be directly involved in genetic rearrangements, which was later corroborated [40,44]. Additionally, *tc*-like genes were identified in various *Xenorhabdus* strains, termed *xpt* genes, and were found to share similar characteristics to the *tc* genes [45]. The discovery of these genetic loci served as the starting point for understanding ABC Tcs. To date, 1421 *tc* loci have been identified in bacterial genomes [4].

**Figure 3:**
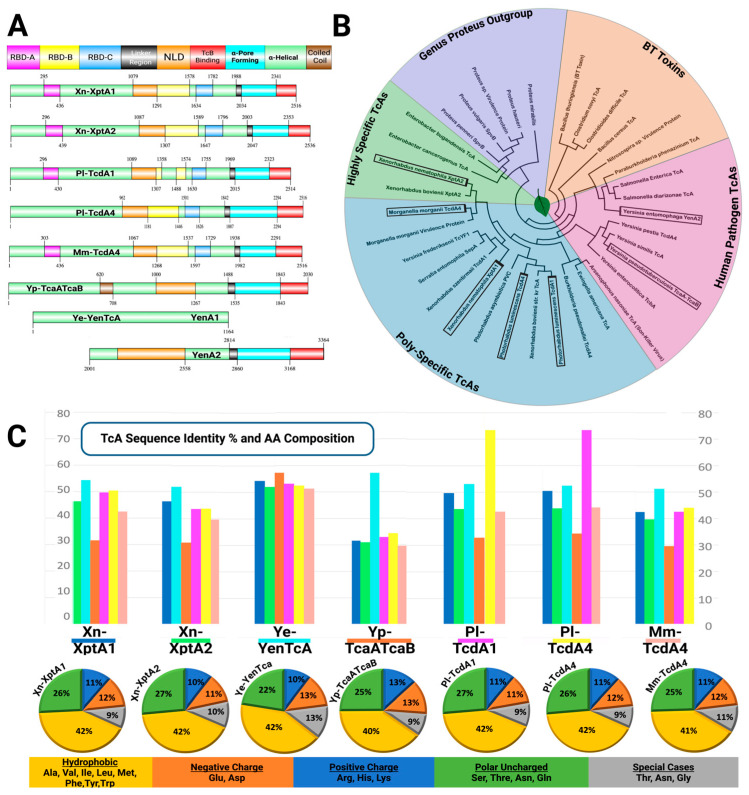
Phylogenetic layout, sequence identity, and domain composition of TcAs. (**A**) Domain layout of the 7 TcAs that have been structurally solved to date. (**B**) Phylogenetic tree grouping a subset of TcAs by specificity and pathogenicity. (**C**) Graphical and pie chart representation of the sequence identity and amino acid composition comparisons between the 7 TcAs that have been structurally solved to date.

ABC Tcs can be divided into two types based off the make-up of the TcA gene copy. Type I, in which five copies of a singular TcA gene form a pentameric assembly, and Type II, where the TcA gene is split in two, in which ten copies of a singular TcA gene form a pentameric assembly [12,20,21]. Piper et al. concludes that the TcA from *Yersinia entomophaga* Ye-YenTcA is a type II TcA due to the split A gene resulting in 10 chains in total for YenTcA pentameric assembly, as opposed to 5 chains total for Pl-TcdA1 [21]. Due to evolutionary gene fusion being four times more common than evolutionary gene fission, we hypothesize that type II ABC toxins preceded type I ABC toxins and that type I ABC toxins resulted from evolutionary gene fusion events [46]. Interestingly the domain layout of both type I and type II Tcs are very similar, sharing high homology between domains and structural composition (Figure 3A). The most heterogeneous domains between species are the RBDs that are responsible for variations in host tropism. Most Tcs can be grouped into different phylogenetic clades based off of targeting specificity (Figure 3B). Here, we outline the seven structurally characterized TcAs based off phylogenetic grouping and their respective sequence identities and amino acid composition (Figure 3C). As expected, the highest sequence identity between all homologues are between TcAs from the same genus and species. Unexpectedly, the type II ABC TcA Ye-YenTcA displayed the highest sequence identity on average compared to the other six characterized Tcs, sharing more than 50% sequence identity across homologues, contributing to the hypothesis that type II ABC Tcs preceded type I ABC Tcs. Overall, the amino acid composition between Tcs varies minimally, showing high similarity between species, with a tight range of hydrophobic amino acids which make up most of the TcAs (40–42%) followed by polar uncharged amino acids (22–27%), negatively charged amino acids (11–13%), and positively charged amino acids (11–13%). Generally, the composition of Tcs are very similar over a broad range of species.

## 3. Structure

**Figure 4:**
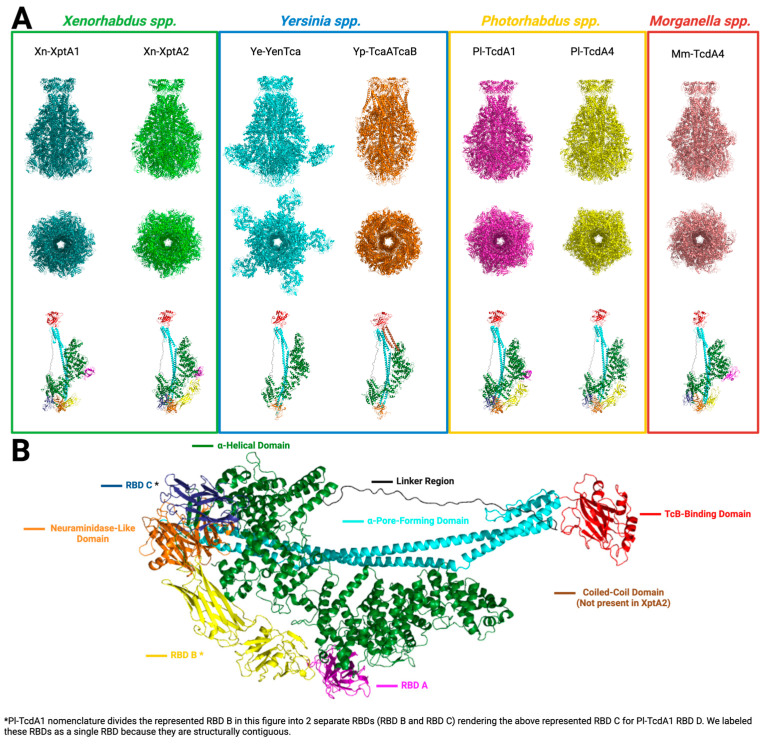
Structural comparisons of TcAs. (**A**) The 7 TcAs that have been structurally solved are depicted in their respective multimeric forms and grouped according to the species of bacteria they are produced in. The depiction shows side views of each TcA as well as bottom views in the second row. (**B**) A single monomer from each protein color coded by domain. The bottom pane displays a representative TcA monomer form *Xenorhabdus nematophila* Xn-XptA2 and serves as a key depicting the color code layout of domains that is represented in all TcAs shown.

The basic structural components of Tcs share many similarities across species. These similarities and subtle differences can be visualized best on the largest subunit of the Tc, the TcA (Figure 4). Some of the earliest structural work with *Xenorhabdus nematophilus*-XptA1 (Xn-XptA1) and *Xenorhabdus nematophilus*-XptA2 (Xn-XptA2) had previously been reported to have tetrameric assembly that assembled in a 4:1:1 stoichiometry [9,47]. More recently, the high-resolution structure of Xn-XptA1 and Xn-XptA2 was solved by both X-ray crystallography and cryo-EM, showing that Xn-XptA2 and Xn-XptA1 form a pentamer, aligning with most of the structural evidence in the field for TcAs [16,21,22]. The most structurally conserved areas between TcAs are the TcB-binding domain (red), the α-pore-forming domain (cyan), the α-helical domain (green), the neuraminidase-like domain (orange), and the linker region (black) (Figure 4). The α-pore-forming domain generally contains anti-parallel α-helices that are inter-connected by loops, leading to the TcB-binding domain on the C-terminal end of the TcA. These α-pore-forming domains come together, forming the translocation channel of the TcA with hydrophobic loops at the very tip of the channel close to the N-terminus. These hydrophobic loops are shielded by the neuraminidase-like domains at the tip of the TcA and are responsible for perforation of the target cell membrane after the TcA undergoes conformational change [19,20]. The neuraminidase domains shielding these loops serve as an electrostatic lock that undergoes destabilization before the pre-pore to pore state transition [19,20]. The linker region is a loose assortment of amino acids that connects the α-pore-forming domain to the α-helical domain and is proposed to be the driving force for conformational change into the pore state [19,20]. The α-helical domain makes up most of the outer shell of the TcA and is interwoven with IgG-like RBDs as well as the neuraminidase-like domains which shield the inner α-pore-forming domains from the outer solvent [20].

To further compare the structural composition between TcA subunits, we analyzed the seven structurally homologous TcAs that had previously been published and deposited in the PDB. Of the TcAs analyzed, two were from *Xenorhadus nematophilus*: Xn-XptA1 (6RW8) [16] and Xn-XptA2 (8TQE) [22]; two from *Photorhabdus luminescens*: Pl-TcdA1 (6RW6) [16] and Pl-TcdA4 (6RWA) [16]; and a single TcA from each of the following: *Morganella morganii*: Mm-TcdA4 (6RW9) [16], *Yersinia pseudotuberculosis*: Yp-TcaATcaB (6RWB) [16], and *Yersinia entomophaga*: Ye-YenTcA (6OGD) [21] (Figure 4). Interestingly, all TcAs display structural homogeneity between the majority of their domains, with the exception of each TcA’s putative RBDs and the addition of a coiled-coil domain in Yp-TcaATcaB.

Most notably, the *Yersinia* Tcs Ye-YenTcA and Yp-TcaATcaB do not have a single putative RBD modeled on the YenA1/YenA2 combined chain or the TcaATcaB monomer [16,21]. The RBDs observed on all other TcAs comprise a consistent mixture of β-sheets and loops that are interwoven between the α-helical domain, making up the majority of the outer shell of the TcA. Additionally, Ye-YenTcA has been shown to form a complex with auxiliary endochitinase domains (Chi1 and Chi2) that make contact with the α-helical domain (Chi2) and an unknown density located near the neuraminidase-like domain [21]. Furthermore, Yp-TcaATcaB also has an unknown density that is not modeled within the same region when structurally aligned [16]. These unknown densities are within the regions of the Ye-YenTcA and Yp-TcaATcaB structures that are missing ~189 amino acid residues (S2333-L2522) and ~99 amino acid residues (N1140-L1239), respectively. Structural alignment of Ye-YenTcA and Yp-TcaATcaB with other TcA orthologs within this region place these missing amino acid residues close to the putative RBD B region: A1310-K1591 for Xn-XptA2 (~281 aa), D1291-L1576 for Xn-XptA1 (~285 aa), I1312-L1576 for Pl-TcdA1 (~264 aa), Y1182-Q1446 for Pl-TcdA4 (~264 aa), and S1268-K1537 for Mm-TcdA4 (~269 aa). These data suggest that the unknown density within Ye-YenTcA may be an RBD that could not be modeled due to the dynamic nature of the domain.

Moreover, the published structures of Xn-XptA1 and Mm-TcdA4 display 160 (S1339-Q1499) and 123 (D1323-M1446) amino acid residues of missing sequence within the RBD B region, respectively [16]. These data indicate that the modeling of these residues is complicated due to a lack of density or poor local resolution within this region. The dynamic nature of TcAs may be responsible for the poor resolution within this region, suggesting that RBD B undergoes structural rearrangement or conformational change. Due to the location of RBD B on the TcA and the proposed dynamic nature, the data suggest that RBD B may be important for host tropism.

To date, the only published structures of the heterodimeric TcB-TcC proteins are the Ye-YenBYenC2 and the Pl-TcdB2-TccC3 structures [25,26,27,48,49]. The TcB subunit is the second largest subunit (~166 kDa) and the TcC subunit is the smallest subunit (~107 kDa) of the full Tc. These subunits are primarily made up of β-sheets and come together to form a cocoon-like structure that contains the toxic peptide termed the hypervariable region (HVR) located on the C terminus of the TcC subunit [27,48]. Along with the HVR, the TcC subunit makes up a large portion of the inner cavity called the “upper chamber”, which binds tightly with the “lower chamber” of the TcB to form an inner area that is largely hydrophobic [48]. Busby et al. suggests that the close proximity of the C-terminus of the TcB to the N-terminus of the TcC indicates that these proteins could be translated as a single polypeptide [27]. Below the lower chamber of the TcB resides a “pre-chamber” area containing a β-propeller with pseudo six-fold symmetry that makes up part of the TcA binding domain [26]. It has been demonstrated that the high affinity of TcB to TcA is due to the complementary hydrophobic and electrostatic interactions across the large interface between the subunits [26]. Interestingly, two of the β-propeller blades (3 and 4) known as the gate-keeper domain undergo conformational change after the full Tc is formed, opening up the channel linking the TcA to the TcB subunit and allowing for continuous passage of the HVR through the TcA [26]. The trigger for this conformational change is caused by the clashing of two sensor loops between the TcA and TcB binding domains of each subunit [26]. To further understand the differences between TcB and TcC homologues, it is necessary to obtain the structures of these proteins both alone and in complex with their respective TcA subunits.

## 4. Binding Interactions and Targeting

Current studies in the field have confirmed that the TcA component of the Tc is responsible for host recognition [20,21,32,47]. Further analyses of Tcs suggest that targeting is driven by one or more of the putative RBDs located on the outer shell of the TcA [21,48]. These RBDs contain IgG-like folds similar to the conserved β-sheets interspersed between loop regions within antibodies, which are commonly associated with cellular binding [50]. Interestingly, the RBDs between TcA homologues are the least conserved domains across TcA species, suggesting that the RBDs are responsible for variability in host tropism [16,51]. The gap between the RBDs and the membrane is ~125 Å, suggesting that the target receptors protrude significantly from the membrane [48,52].

Previously, Sheets et al. showed that the Xn-XptA2 toxin complex XptA2-XptB-XptC displayed targeting to the *Helicoverpa zea* midgut brush border membranes, having a 1:1 interaction with the immobilized ligand, although a specific receptor was not identified [9]. Roderer et al. shows that Pl-TcdA1 can perforate membranes in the absence of a receptor, indicating that the Tc may not require a binding partner if the pre-pore to pore state transition occurs in close proximity to the cell membrane [25]. It is noteworthy to add that Pl-TcdA1 displayed a preference for zwitterionic lipids found in insects and mammals over negatively charged lipids commonly found in bacteria [25]. To our knowledge, only one protein receptor for any TcA has been identified [51]. Pl-TcdA1 has been shown to bind to the *O*-glycosylated mucin-like domain of Visgun (Vsg) at sites with repetitive proline, threonine, and serine (PTS) residues [51]. Vsg orthologs that do not contain these repetitive PTS repeats along this domain are not susceptible to Pl-TcdA1 Tc intoxication [51]. Xu et al. displays the additive effects of Pl-TcdA1 on binding when Vsg contains *O*-glycans. Although the data suggest that *O*-glycans increase the binding interaction of Pl-TcdA1 to Vsg, they are not necessary for binding to occur [51]. Additionally, Xu shows that *N*-glycans have no observable effect on Pl-TcdA1 binding to Vsg, suggesting a glycan-specific mode of targeting to Vsg [51].

Although the specific role of cell surface glycans in Tc binding is not clear, current evidence suggests that interaction with these glycans may be a prerequisite of TcA host cell recognition [21,51,53,54,55]. Roderer et al. showed that differences in complex cell surface glycans contribute to the binding of Pl-TcdA1 to mammalian cells [54]. A glycan microarray was performed on Pl-TcdA1, Xn-XptA1, Mm-TcdA4, and Yp-TcaATcaB, displaying that the Lewis X glycan (found on both *N* and *O*-linked glycans) binding interaction is specific for Pl-TcdA1 due to the lack of interactions with any of the other aforementioned TcAs [54]. Additionally, the glycosaminoglycan heparin was shown to bind to Xn-XptA1 and Mm-TcdA4 [54]. Structural analyses of these glycans bound to each TcA reveal that the Lewis X antigen binds Pl-TcdA1 at RBD D, while heparin binds Xn-XptA1 in a gap between the neuramindase-like domain, RBD B and RBD D, and Mm-TcdA4 at the α-helical domain near the mid-line of the TcA [54]. These data show the unique modes of binding between TcA orthologs and how TcAs mediate cell surface recognition.

Furthermore, most TcAs contain “lectin like” domains in the form of neuraminidase-like domains and, in some cases, auxiliary chitinase domains. It has been proposed that some Tcs such as Ye-YenTcA may require auxiliary chitinase domains to facilitate targeting to the host cell [12,21,56,57]. Chitin is the second most common polysaccharide in nature behind cellulose and is found in abundance within the digestive tract of insects [58]. Landsberg et al. was the first to identify a TcA, Ye-YenTcA, as an endochitinase [12]. Building off of this work, Piper et al. shows that Ye-YenTcA and the chitinase domains that form the Ye-YenTc, Chi1 and Chi2, bind to a host of glycans containing galactose, glucose, N-acetylgalactosamine, and N-acetylglucosamine motifs [21]. Ye-YenTc alone displayed a higher prevalence of binding to fucosylated structures and mannosyl derivatives, both of which are numerous in insects, suggesting a mechanism for Ye-YenTc insect host tropism [21]. Moreover, Ye-YenTc insect specificity was further validated by the narrow range of binding to complex *N*-linked glycans and sialylated structures common in vertebrates [21].

Alternatively, Pl-TcdA1 Tc has been shown to target various insect and mammalian cell lines, indicating a broader range of target cell specificity [26,29,53]. Song et al. demonstrates that disrupting the production of *N*-glycans in HeLa cells through KO and pre-treatment with kifunensine, an inhibitor of class I α-mannosidases that inhibits the synthesis of *N*-glycans, significantly inhibits the toxicity of Pl-TcdA1 Tc, indicating the importance of *N*-glycans for Pl-TcdA1 Tc cell surface recognition to HeLa cells [53]. Moreover, Song indicates that a KO of the *O*-linked glycan core 1 glycoprotein-*N*-acetylgalactosamine 3-β-galactosyltransferase 1 (C1galT1) had no effect on inhibiting cell intoxication from Pl-TcdA1 Tc, further suggesting broad-range glycan-specific recognition when considering previous reports demonstrating additive effects on binding to the *O*-linked glycans on Vsg [51,53].

When Pl-TcdA1 (note that Pl-TcdA1 used throughout this review refers to the W14 strain) was compared to Pl-TcdA1 TT01, it was shown that KO or inhibition of N-glycan synthesis had no effect on the TT01 homologue from subspecies *Photorhabdus laumondii* [53]. When analyzed in further detail, it was discovered that the highest contrast in the 88% sequence identity between both TcAs lay within RBD D, supporting the conclusion that the RBD D of Pl-TcdA1 is responsible for host tropism through N-glycan interactions [53,54]. Further solidifying this conclusion, Song et al. showed that swapping the RBDs through genetic modification decreased the Pl-TcdA1 Tc TT01 strain’s ability to intoxicate *N*-glycan-deficient HeLa cells, while the modified Pl TcdA1 Tc strain’s ability to intoxicate *N*-glycan deficient cells was revived [53].

Song also demonstrated that Pl-TcdA2 Tc TT01 intoxication was mediated by the sulfate group on sulfated glycosaminoglycans (sGAGs) by pre-treating HeLa cells with the small molecule surfen, an antagonist of heparin binding, before seeing a marked decrease in toxicity [53]. It is not uncommon for charged sulfate groups to be the target of microbial toxins. For example, *C. Difficile* and *H. Pylori* both have cytotoxins that interact with charged sulfate groups [59,60]. Likewise, Roderer et al. demonstrates that Xn-XptA1 and Mm-TcdA4 interact with heparin sulfates through direct cryo-EM analysis of the binding interaction [54]. Although an interaction with the charged sulfate groups is clear in some TcAs, other TcAs such as Yp-TcaATcaB, Pl-TcdA1 W14, and TT01 do not target charged sulfate groups [53,54].

Previous work also shows that the binding specificity and potency of Pl-TcdA1 significantly increases when the TcA is cleaved by proteases yielding cleavage products that range from 10 to 200 kDa [61]. Processing of Pl-TcdA1 with various proteases seemed to have no effect on pore formation and demonstrates an increased binding specificity, although further exploration of these data is needed to determine the mechanism by which these occur [61]. Building off of this work, Ng’ang’a et al. performed a glycan microarray with protease-exposed and non-protease-exposed Pl-TcdA1 and confirmed that the top hits between all Pl-TcdA1 isoforms were *N*-glycans, although marked differences in the glycan binding profile were observed between cleaved and uncleaved Pl-TcdA1 [55]. Cleaved Pl-TcdA1 exhibited two-fold higher relative fluorescent unit values, demonstrating that the cleaved Pl-TcdA1 had a higher binding affinity than uncleaved Pl-TcdA1 [55]. Additionally, Ng’ang’a presented an in silico analysis predicting the binding sites of various N-glycans and, unsurprisingly, the top hits were across all of the putative RBDs on Pl-TcdA1, with multiple RBDs containing multiple N-glycan binding sites [55]. Taken together, these data substantiate the conclusion that multiple different RBDs across TcA homologues are involved in TcA targeting. Furthermore, glycans serve as a binding partner for many TcAs and contribute to the targeting variability between TcAs.

## 5. Translocation Mechanism

The plasma membrane consists of a bilayer of amphipathic phospholipids made up of hydrophilic phosphate head groups as well as hydrophobic acyl chains, creating a barrier between the cytosol and extracellular space [62]. Most biological membranes vary in width between 3 and 10 nm depending on the lipid content and membrane protein composition [63,64]. The plasma membrane of both eukaryotes and prokaryotes are the first line of defense from external threats associated with pathogenicity, such as pore-forming toxins (PFTs) [65]. PFTs belong to the largest class of bacterial toxins that target the plasma membrane and can be divided into two classes: α-PFTs, consisting of a pore-forming domain primarily made up of α-helices, and β-PFTs, consisting of a pore-forming domain primarily made up of β-sheets [65,66]. ABC Tcs are α-PFTs consisting of a heterotrimeric complex between three subunits (TcA, TcB, and TcC) that perforate biological membranes for the purpose of injecting toxic components into the target cell [16,20,21].

After the TcA component comes into close proximity to the cell membrane, it has been shown to undergo a pH/mechanical stress-dependent conformational change in which the outer shell of the TcA slides down the translocation channel and rearranges to partially encapsulate the TcB-TcC subunits [19,21,25,48]. Exposure to very acidic and basic conditions causes a repulsion of the neuraminidase-like domains, shielding the transmembrane region of the translocation channel, which suggests that this region serves as an electrostatic lock [48]. Upon structural rearrangement into the pore state, three major hinge regions within the RBDs and α-helical domain have been identified in Pl-TcdA1 and are necessary for the outer shell to unfold [48]. Previous work has proposed that α-helical proteins perforating the cell membrane lie parallel to the membrane prior to insertion [67]. Contrary to this proposed mechanism, TcAs have been shown to perforate membranes in a perpendicular orientation after the pre-pore to pore state transition [19]. Gatsogiannis et al. proposes that the driving force of this conformational change is a largely disordered linker region that contracts into a partially folded α-helix upon pore-state activation [19]. Leidreiter et al. showed that all TcAs have a conserved 3_1_ trefoil protein knot beginning at the base of the linker region, finding that TcAs are the largest proteins to contain a trefoil knot [16]. It is suggested that this trefoil knot stabilizes the TcA at the base of the linker and is important for maintaining the structural integrity of the TcA upon conformational change. Although it is important for conformational dynamics, it has been demonstrated in Xn-XptA2 that a continuous linker region is not necessary to produce fully folded pre-pore TcA [22]. Apart from the linker region, the other domains making up the TcA are not significantly altered during the pre-pore to pore state transition [48]. It is proposed that this entropy-driven contraction of the linker region is the driving force for conformational change into the pore state and is responsible for the “syringe like” injection of the toxic peptide across lipid bilayers [48].

The TcB-TcC subunits form a cocoon that binds to the TcA through the TcB β-propeller in a 5:1:1 stoichiometric ratio [26]. Upon binding, the β-propeller undergoes a conformational change in which the TcC cavity is opened, allowing the toxic peptide to thread into the TcA translocation channel [26]. The hydrophobic residues lining the inside of the TcB-TcC cocoon house the unfolded toxic peptide located on the C terminus of the TcC protein, known as the hypervariable region (HVR) due to its sequence variability across TcCs [29]. This ~30 kDa HVR is autoproteolytically cleaved and linearized through the β-propeller into the TcA, where it is subsequently translocated into the target cell upon binding and conformational change [27,48]. Interestingly, Roderer et al. shows that the TcB-TcC subunit can form the holocomplex and translocate the HVR into the TcA after the TcA has perforated the membrane, suggesting that toxin translocation is not driven by an external energy source [25]. Additional studies have also shown that the TcB-TcC subunit displays a decrease in affinity to the TcA subunit when no cargo is present or when the size of the cargo is below a certain threshold (~20 kDa) [26,28]. When taken together, these data suggest that following translocation, the affinity of TcB-TcC to TcA significantly decreases and may disassociate from the complex, leaving the opportunity open for a separate TcB-TcC to bind to the imbedded TcA.

**Figure 5:**
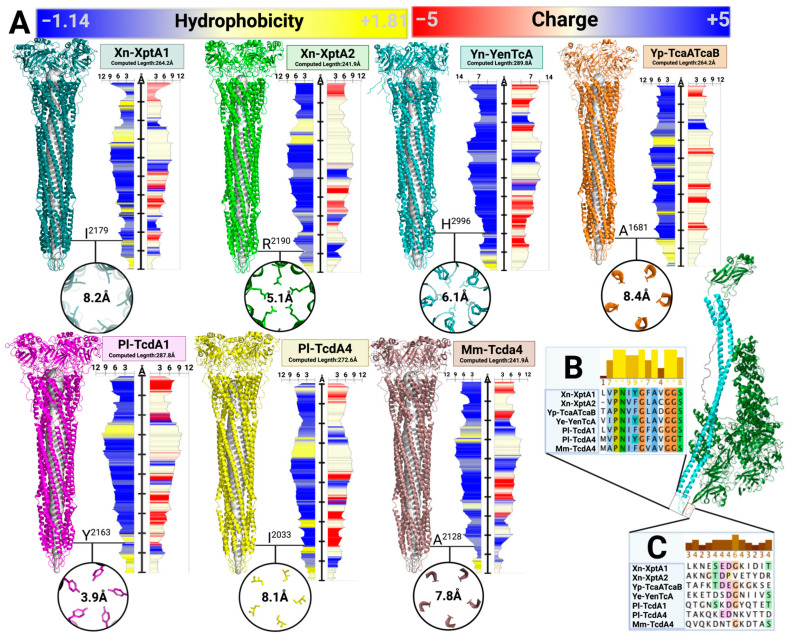
Analyses of translocation channels, pore-forming loops, and pore-closing loops between TcAs. (**A**) Comparison of all TcA translocation channels measuring the channel density and highlighting the most constricted region of the channel apart from the pore forming loops. (**B**) Pore-forming loop amino acid sequence conservation between TcAs. (**C**) Pore-closing loop amino acid sequence conservation between TcAs.

The translocation channel of Pl-TcdA1 contains many negatively charged residues that are oriented inwards, indicating that the channel is cation selective [48]. Furthermore, the cationic HVR has been shown to interact with hydrophobic and negatively charged regions of the translocation channel [25]. A comparative analysis between the translocation channels of the seven structurally solved Tcs shows a similar trend in which most of the residues are hydrophilic and negatively charged with small hydrophobic and positively charged patches of amino acids dispersed throughout the channel (Figure 5A). These data indicate that peptide motility throughout Tcs shares a similar mechanism. Previous work suggests that the overall charge distribution accounts for changes in ion permeability across lipid bilayers [16]. Furthermore, the most constricted site apart from the pore-forming loops seems to reside in a similar area just above the pore-forming loops (Figure 5A). Although the residues at this site vary between all seven Tcs, the diameter of these constriction sites is fairly uniform, with a range from 3.9 Å to 8.4 Å for an average diameter of 6.8 Å across constriction sites. It is unclear how these sites differ when induced into the pore-state conformation due to a lack of TcA structures in the pore-state conformation.

The pore-forming loops on the end of the α-pore forming domains that make up the end of the translocation channel are highly conserved across homologues (Figure 5B). Most of these residues are hydrophobic, which creates an energetically unfavorable environment when exposed to the intracellular hydrophilic environment. This causes the pore forming loops to stabilize in the nearest amphipathic membrane, resulting in pore formation across the membrane. The high conservation of residues in this region indicates that this mechanism is uniform across TcAs. The pore-closing loops on the neuraminidase-like domains shield the pore forming loops from the external environment until conformational change occurs. It is proposed that this region may be an electrostatic lock making contacts with adjacent protomers from the TcA, becoming destabilized under extreme pH conditions. The amino acid sequence conservation in this region is very low across TcA homologues, suggesting that this region could be responsible for the variability in pH-dependent conformational dynamics (Figure 5C). Although this region is of clear importance for TcA dynamics, additional work is needed to fully understand and test this hypothesis.

## 6. Toxicity

Early studies regarding the insecticidal activity of TcAs suggest that the TcA subunit alone has cytotoxic effects on target organisms and is greatly enhanced by the addition of the TcB and TcC subunits [32,68,69]. Xn-XptA2 exhibits modest oral toxicity to lepidopteran insects, with toxicity increasing significantly after the formation of the full toxin complex [9]. Morgan et al. found that Xn-XptA1 and Xn-XptA2 had little to no toxic effect on *P. brassicae* and that Xn-XptA1 in complex with the TcB and TcC subunits increased toxicity to *P. brassicae* larvae. Interestingly, Xn-XptA2 in complex with the TcB and TcC subunits was not toxic to the *P. brassicae* larvae [45]. These data suggest that TcAs within the same species can display variations in host tropism and confirm that a full ABC complex is necessary to achieve toxicity to the target organism.

Most characterized Tcs have shown potent toxicity to insects, although the range of insects targeted vary greatly between homologues. ABC Tcs from *X. nematophilus* display toxicity to *Lepidoptera* species, with Xn-XptA1 Tc displaying a broader range of *Lepidoptera* host tropism than the Xn-XptA2 Tc [9,47]. Alternatively, *P. luminescens* Tcs display a broader range of oral insect toxicity to *Lepidoptera*, *Coleoptera*, *Dictyoptera*, *Leptinotarsa*, *Decemlineata*, and *Bemisia* species [2,3,24,70]. Another broad-range Tc includes the *Y. entomophaga* YenTc, which demonstrates a wide range of toxicity to *Lepidoptera* and *Coleoptera* species [6,12,27,30,71]. Interestingly, some Tcs display toxicity across biological classes, with Tcs from *Photorhabdus* and *Yersinia* species displaying toxicity in vitro to various mammalian cell lines. Meusch et al. shows that Pl-TcdA1 in combination with Pl-TcdB2-TccC3 has a toxic effect on HeLa cells, causing actin polymerization within the cells that can be viewed through fluorescent microscopy [48]. In addition to HeLa cells, Pl-TcdA1 Tc has been shown to have cytotoxic effects on HEK293T cells and CHO cell lines [53,54,55]. These data indicate that Pl-Tcs have evolved over time to be pathogenic to mammals. Likewise, other Tcs found in *Yersinia pseudotuberculosis*, *Yersinia pestis*, and *Morganella morganii* have shown an adapted pathogenicity to mammals [7,36].

**Figure 6:**
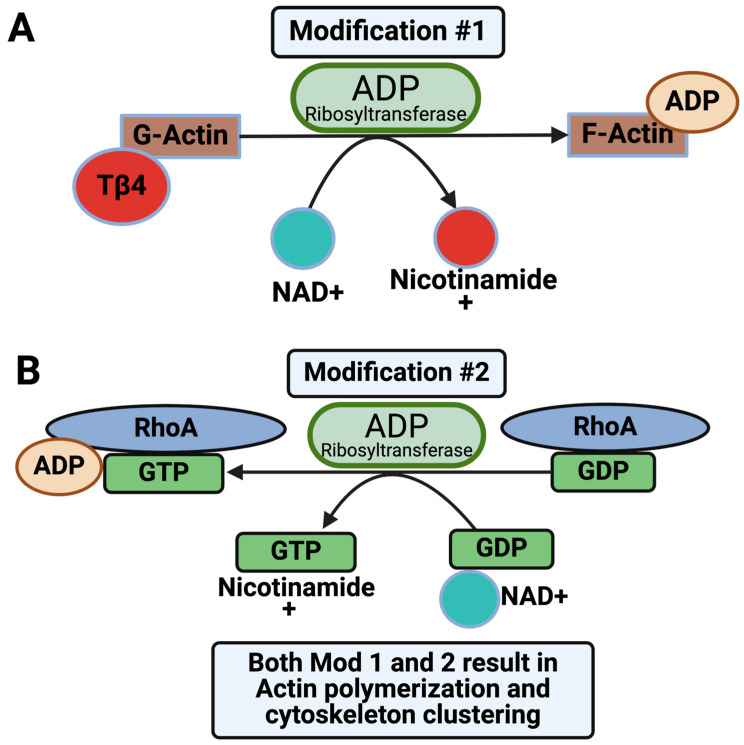
TcC mechanisms of cytotoxicity. (**A**) TccC3 ADP ribosylates G-actin, inhibiting the actin thymosin–β4 interaction, resulting in G-actin sequestration and actin polymerization. (**B**) ADP ribosylation of RhoA inhibits GTP hydrolysis, rendering the GTP-binding protein obstinately active.

The peptide responsible for toxicity in the ABC Tc is located within the HVR on the C-terminus of the TcC. This HVR is autoproteolytically cleaved and refolds within the cytosol, functioning as an ADP ribosyltransferase (TccC3) or Rho GTPase (TccC5) encapsulated within the TcB-TcC subunits [9,12,18,28,29,48]. Pl-TccC3 is shown to ADP-ribosylate threonine 148 of actin, where actin is known to interact with the actin-binding protein thymosin-β4 [29]. This binding interaction results in G-actin sequestration, thus preventing the inhibition of actin polymerization, leading to cytotoxicity [29,72] (Figure 6A). Pl-TccC5 ADP-ribosylates Rho and Rac proteins at glutamine 61 and 63, respectively, which are regions that stimulate GTP-hydrolysis [29,49,73]. This binding interaction inhibits GTP hydrolysis and renders the GTP-binding protein obstinately active, causing the formation of stress fibers within the cell leading to cytotoxicity [29] (Figure 6B).

Interestingly, chitinases can be found in similar locations close to Tc loci across various species, including but not limited to *X. nematophila, Y. entomophaga*, and *P. luminescens* [56]. Previous work has identified the necessity of chitinases for the insecticidal activity of Ye-YenTc [6,12]. The chitinases found near the Tc loci in *X. nematophila* display insecticidal activity in the absence of the Tc [56]. Liu et al. suggests that these chitinases are vital to the insecticidal activity of *X. nematophila* Tcs, although previous work displays potent oral toxicity to lepidopteran insects without the addition of chitinases [9,32]. Chitinases are essential for some Tcs to induce toxicity, while other Tcs show that chitinases are not necessary for Tc cytotoxicity. It is clear that the role of chitinases across Tc homologues is understudied and presents an opportunity to understand the functional differences between these auxiliary domains across homologues.

## 7. Biotechnology Applications

**Figure 7:**
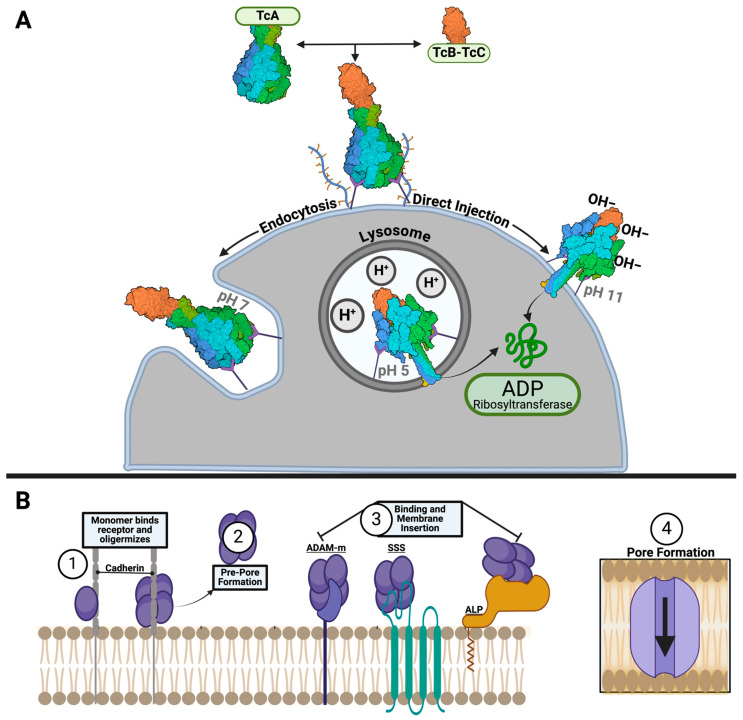
Comparison of ABC toxin mechanism to Bt toxin mechanism. (**A**) Schematic representation of the two proposed mechanisms for ABC toxin binding and cytotoxicity. (**B**) Schematic representation of Bt Cry toxin assembly and membrane permeation.

The innate insecticidal capabilities for many ABC Tcs make them excellent candidates for use in transgenic plants. Currently, *Bacillus thuringiensis* (Bt) toxins are the most common proteins integrated into transgenic plants to control insect pests [74,75]. Although Bt toxins have proven to be effective against many insect pests, a trend in insect resistance has been observed and is a concern for global food production and security [76,77,78]. ABC toxins and Bt toxins are both PFTs, with Bt toxins being studied much more extensively, having practical uses in transgenic crop production since 1938 [79]. Although ABC toxins are primarily compared to Bt toxins, both toxins display distinct differences in their respective mechanisms of cytotoxicity. Bt toxins undergo oligomerization after initial binding to the cadherin receptor, initiating the pre-pore formation of the Cry toxin complex [80]. Once the protein is cleaved by proteases, it is activated from the inactive state to the active state and has been shown to bind to secondary receptors undergoing pore formation and inducing cytotoxicity through disruption of the cell membrane [81,82]. In contrast, ABC toxins assemble before binding to the target receptor and undergo an inactive to active conformational change through a pH gradient [17] (Figure 7A,B). Liu et al. demonstrated that Tc components from *P. luminescens* (W14) can be put into the genome of *Arabidopsis thaliana* and confer insecticidal activity rivaling the LD50 of Bt toxins [83]. Furthermore, insecticidal activity across many different Tcs has been observed for a host of insect pests across the world [1,34,84]. The potential benefit of Tc insecticidal activity has been recognized by Dow Agrosciences, and in 2009 and they patented *Xenorhabdus* proteins and genes for pest control (Patent No.: US 7,517,956 B2).

Alternatively, the unique mechanism of ABC Tc translocation has drawn translational interest as an unconventional targeted drug delivery agent. Roderer et al. shows that the natural mechanism of the Tc could be modified to target and translocate cargo directly into cells. Previous work with Pl-TcdA1 Tc has identified that the cargo within the TcC subunit can be interchangeable, rendering the full ABC complex a potential cargo-carrying shuttle that can inject a range of proteins into target cells [28]. Furthermore, the characteristics of the potential substitutions for the hypervariable region (HVR) were identified, enabling substitutions within this region while maintaining a functional Tc with translocation capabilities. In order to qualify as a translocation protein, the substitution protein cannot have amphipathic helices or large hydrophobic regions that may interact with the inner region of the cocoon. Additionally, this protein must be between 20 and 35 kDa and have a PI of 8.0 or higher [28]. Ng’ang’a et al. displayed similar results, showing the versatility of the Pl-TcdA1 Tc by substituting the hypervariable region of TccC3 with *Clostridium botulinum* ADP ribosyltransferase (C3bot) and YopT from *Yersinia enterocolitica* modified to have a more basic PI [35]. The exposure of these hybrid complexes to Caco-2 and HeLa cells resulted in characteristic morphology changes that occur when the cells die, such as clumping [35]. These data imply a wide range of utility that can be potentially harnessed through the natural cargo-carrying and injection mechanism of ABC Tcs.

Although PFTs in targeted drug delivery and recombinant protein transport across membranes have been explored previously with binary toxins, the unique mechanism of ABC Tcs makes them a viable candidate for a peptide delivery agent, alone or in combination with binary toxins [85,86,87,88]. Zahaf et al. showed that the hypervariable region of the *Photorhabdus luminescens* TccC3 could be coupled to a modified protective antigen (PA) and inert lethal factor (LF), components of the binary anthrax toxin, to present cytotoxicity toward esophageal adenocarcinomas (EACs) and esophageal squamous cell carcinomas (ESCC) [33].

Most hybridizations of Tcs to date have focused on the TcC component or substitutions thereof. Based on the current literature, modifications of ABC TcAs for the purpose of targeting alternative epitopes may be advantageous compared to current delivery agents. The amount of RBDs per chain imply an increase in specificity, although which RBDs or how many are responsible for binding to the host cell/target receptor is still poorly understood. Additionally, the solvent-accessible surface area of the cargo itself is shielded from the outside environment, potentially protecting the cargo from degradation. To further the translational outlook of ABC Tcs, much work still needs to be conducted to build on the progress that has been made thus far. In vivo toxicity studies in mammals and proof-of-concept studies for altering targeting specificity, among other studies, are necessary to understand the complex mechanism of ABC Tcs. Nonetheless, the previous work has paved the way to utilizing this class of toxins for translational purposes.

## 8. Conclusions

ABC toxins are a new class of protein toxins with a novel mechanism for intoxicating target cells. The TcA or targeting subunit contains multiple RBDs per chain, indicating a high specificity for target cell receptors. The pre-pore state allows for shielding of the toxic HVR until activation occurs through mechanical stress, such as RBD contact with the receptor or a pH gradient. The unique translocation mechanism allows for peptide translocation directly across lipid bilayers. The distinctive characteristics of these toxins make them logical candidates as potential protein shuttles for therapeutic applications. Furthermore, the natural insecticidal properties of ABC Tcs demonstrate that these proteins may be of use in transgenic plants as biological pesticides. Although much progress has been made in characterizing ABC Tcs, there are still many unanswered questions that need to be addressed. It is still unclear what protein receptor most TcAs bind to. Furthermore, high-resolution structures of the pore state TcAs and TcAs in complex with TcB and TcC subunits are lacking, making comparisons between homologues difficult. Additionally, further genetic alterations between the RBDs of homologues could provide new avenues for altering target cell specificity. Nevertheless, our current understanding of the structure and mechanism of Tcs has shed light on a unique protein translocase with many potential applications.

## Data Availability

The data presented in this study are available in Pubmed Central. These data were derived from the following resources available in the public domain: https://www.ncbi.nlm.nih.gov/pmc/ (accessed on 15 September 2024).
